# Psychological issues and construction of the mother-child relationship in women with cancer during pregnancy: a perspective on current and future directions

**DOI:** 10.1186/s40359-018-0224-5

**Published:** 2018-03-16

**Authors:** Federica Ferrari, Flavia Faccio, Fedro Peccatori, Gabriella Pravettoni

**Affiliations:** 1 Applied Research Division for Cognitive and Psychological Science, European Institute of Oncology, Via Ripamonti 435, 20141 Milan, Italy; 2 Department of Oncology and Hemato-Oncology, University of Milan, Via Festa del Perdono 7, 20141 Milan, Italy; 3 Fertility and Procreation Unit, Gynecologic Oncology Division, European Institute of Oncology, Via Ripamonti 435, 20141 Milan, Italy

**Keywords:** Cancer, Pregnancy, Mother-child relationship, Mothers, Psychological factors

## Abstract

**Background:**

Cancer during pregnancy is a rare event. However, knowledge about treatment has progressed in recent years with improved maternal and neonatal outcomes. The number of women who decide to continue their pregnancy and undergo cancer treatment is increasing.

**Main body:**

Women face two critical events simultaneously; oncological illness and pregnancy, with different and conflicting emotions. In addition, the last trimester of gestation sets the ground for construction of the mother-child relationship, which is of great importance for the child’s development. Studies have showed that maternal exposure to stressful events during pregnancy is linked to adverse outcomes in children. Although several authors consider cancer to be a ‘critical life event’, studies that address the psychosocial implications of cancer in expecting mothers are scarce. There are no studies addressing the possible negative impact of a cancer diagnosis during pregnancy on the mother-child relationship and on the child’s development. It is important to emphasize the need for in-depth knowledge of the contributing psychological factors involved in order to provide holistic, individualised, and supportive care.

**Conclusion:**

An analysis of cognitive aspects, emotional processes, and maternal attachment in cases of cancer during pregnancy may contribute to the development of a model of care, both in an evolutionary and in a psycho-oncology context, with implications for clinical practice.

## Background

Cancer during pregnancy is relatively rare, with an estimated incidence of 1/1000–2000 pregnancies [1]. However, this incidence rate will probably rise in the next ten years due to a higher percentage of women delaying pregnancy until their late thirties and early forties – an age group in which cancer diagnoses are increasing [2]. Another contributing factor might be the increased frequency of obesity, the improvement of diagnostic techniques, and heightened awareness of the importance of screening and self-examination [3]. Other unknown factors are probably at play, and these should be explored so as to increase mothers’ chances of survival and improve neonatal outcomes.

Historically, cancer during pregnancy has had a worse prognosis than non-pregnancy associated cancer. For this reason, the medical indication was to terminate the pregnancy [4]. Currently, the survival rate of pregnant and non-pregnant patients with cancer is similar, and pregnancy per se does not worsen the prognosis of the disease. Moreover, it seems that cancer does not impact negatively on the child’s cognitive and cardiac development [5]. The most frequently encountered tumor types are breast, hematologic and dermatologic cancers [2], while an epidemiologic study conducted in 2012 in the US also encountered several cases of cervical cancer, second only to breast cancer [6], followed by leukemia and dermatologic cancers.

Differently from other cancers, breast cancer during pregnancy can show a diagnostic delay ranging from 2 to 15 months [1], which is partly due to the woman’s primary focus on pregnancy and to the misinterpretation of breast cancer symptoms, which are attributed to pregnancy and breastfeeding [7]. The treatments recommended by international guidelines are surgery, which can be performed safely at any time during pregnancy, and chemotherapy, which can be administered during the second and third trimester [4]. On the other hand, radiotherapy treatment remains questionable [1, 5].

A woman with cancer during pregnancy faces a real paradox, as pregnancy is a symbol of new life, while cancer is a potential threat to her life and that of her child. On the one hand, patients can express feelings of hopelessness, fear and anxiety related to their illness and concerns about the ability to sustain the pregnancy [8]. On the other hand, they manifest joy for bringing a new life to the world and they demonstrate a fighting spirit, supported by the desire to be present in their child’s life [7]. The quality of the relationship with their partner and a stable environment are important resources that contribute to the patient’s ability to cope with the diagnosis and treatment plans [7].

For these patients, the decision-making process with regards to the course of pregnancy and cancer treatment is of particular importance. The patient has to make highly emotional decisions involving her own wellbeing, but also that of her fetus, in a short time [9, 10]. Given the emotional burden that the patient – or couple - must endure, it is imperative to evaluate and address their concerns, provide clear information about the disease and, in a shared-decision making context, discuss treatment plans and the continuation or interruption of the pregnancy [11]. It is essential to empower the patient by communicating the risks of each option and providing clear and precise medical information [12]; this allows the patient to make, in a short time frame, an informed decision regarding the impact on their own health and that of their unborn child [13].

While various researchers have highlighted the importance of investigating psychological aspects in women with cancer during pregnancy [1, 7], these have not been explored sufficiently. One of the aspects which has been overlooked is the development of the mother-child attachment in expecting mothers with cancer. It is therefore important to increase knowledge about the contributing psychological factors in order to provide integrative, individualised, and supportive care.

## Main text

### Cancer during pregnancy and its influence on the mother-child relationship

Pregnancy is considered to be both a developmental stage and a stage of extreme vulnerability for women [14]. Here physical and psychological changes occur, such as the psychological process that prepares the mother-to-be for her new responsibilities [7]. From the tenth week of gestation it is possible to see the raw emotions typical of attachment system; these grow more intense after the first fetal movements and more generally in the last trimester of pregnancy [15]. The mother-child relationship begins with the mental representation of the foetus and continues with the construction of an emotional bond based on the search for caregiving [14, 16]. It is important to emphasize that this bond can influence from the very beginning the child’s neuropsychological, emotional, and relational development [17]. One of the main developmental steps during pregnancy is the costruction of the prenatal attachment. If this developmental task is not accomplished, both the mother and her child may experience poor emotional adjustment and may encounter significant difficulties in the establishment of the mother-child relastionship after birth [14, 17]. Studies measuring prenatal attachment through questionnaires and subsequent postnatal attachment through observations of mother-child interactions, have noticed that mothers who scored higher in prenatal attachment measures showed more involvement and more frequently stimulated their infants compared to those mothers who perceived less emotional involvement in the last trimester of pregnancy [18, 19]. However, there are strong contributing factors, such as the presence of depressive symptoms, that can negatively impact on both prenatal attachment and the mother-child relationship during the post-partum period [17, 20, 21].

Numerous studies have showed that maternal exposure to stressful life events during pregnancy is associated with preterm birth, low birth weight and neurodevelopmental impairments [17]. Indeed, the mother may be physically and emotionally less able to build a relationship with her child and take care of him/her in the postpartum period; this can be said also for expecting mothers with cancer. As Elmberger and colleagues [22] emphasize, mothers with cancer often struggle to maintain their parental role and to function as “good mothers” due to their limited availability and exhaustion. This experience might impact on the construction of the mother-child relationship [23].

### Current knowledge about psychological aspects of cancer diagnosis during pregnancy

To date, few studies have addressed psychosocial implications of cancer in expecting mothers and they present significant limitations. Through the use of self-administered questionnaires, Henry and colleagues [9] showed that patients with gestational cancer are likely to manifest clinically significant levels of distress if they experience cancer recurrence or risk of preterm birth, if they are advised to terminate the pregnancy, or if they have to undergo surgery during pregnancy [9]. This study recruited a small sample, and time between diagnosis and questionnaire administration was different for each patient. While psychological measures in proximity to diagnosis communication are more sensitive to the level of distress, measuring psychological symptoms after the event can inform on long-lasting influences.

Another study used a qualitative approach and investigated emotional and social issues retrospectively [13]. The main themes that emerged from the interviews were anxiety and distress throughout the whole duration of the pregnancy and after the child’s birth. In particular, anxiety was primarily linked to the patient’s concern over their limited availability and their baby’s health [13].

Finally, a study attempted to profile patients with gestational cancer and their partners [24]. The authors noticed that patients and their partners exhibited similar levels of distress, and that patients who used internalizing coping strategies had higher levels of concern for their child’s health, the disease and the treatment plan, and were therefore at higher risk of showing clinical distress [24]. However, they administered a non-standardized questionnaire, developed specifically to measure the psychological burden of cancer during pregnancy.

Due to the scarcity of studies it is possible to infer that the challenging situation in which the cancer diagnosis is communicated to the patient, namely pregnancy, may be overlooked or underestimated. Moreover, some parents-to-be are not embedded in a supportive social network and this can lead to increased levels of distress. A multidisciplinary team can help these couples by activating emotional and practical support, which would allow them to adjust to treatment plans and sustain their role and responsibility as a parent [22].

### Future directions

There are several issues concerning the psychological management of gestational cancer that should be addressed; one of these is the degree of explanation these women receive from their oncologist with regards to the risks and available treatments. Another related aspect is whether they are allowed to take on an active role in the delicate decision-making process that might affect their own health and/or that of their unborn child. Standardised questionnaires and short, semi-structured interviews can capture these issues, which can then be raised in multidisciplinary clinical meetings in order to improve doctor-patient communication, reduce influence of personal prejudices, and respect the decision made by the patient.

Social support and emotional regulation, which are crucial aspects in non-expecting patients, should be addressed in mothers-to-be and inter-group comparisons should be conducted. While cancer patients under 45 years of age are psychologically more vulnerable than older ones due to the impact on current/future relationship with the partner, fertility, and uncertainty about the future [6], pregnant counterparts may show resilience in adjusting to illness-related changes, driven by a fighting spirit for their child’s and their own wellbeing. It is of paramount importance to provide a psychological evaluation of quality of life and psychological functioning of the parents-to-be, which carefully analyses anxious-depressive symptoms, the dyadic relationship of the couple, their support network, and the impact of the oncological disease on their lives. An early assessment of parents’ psychological well-being during pregnancy, including an evaluation of the maternal attachment style, would allow for the identification of “at risk” individuals, who can be referred to perinatal and postnatal support to prevent adverse pychological outcomes. Moreover, it may be helpful to develop and test an evidence-based psychological intervention that can promote parents’ resources to deal with the difficulties they encounter. Within this intervention, support from different professional figures can be activated in parallel to the psychological one, on the basis of the specific needs of the family at different stages of their therapeutic pathway [25].

As the oncologist’s primary focus with cases of gestational cancer is the treatment and survival of the patient, some psychological aspects may be overlooked. As a holistic approach to patient care is recommended, it is vital to understand the emotions and concerns of these women in order to increase awareness and knowledge about the type of support that should be offered in oncological settings. As these patients often report feeling “different” from other mothers-to-be, practical and emotional peri-natal support, based on empirical evidence, can help women feel less isolated and allow them to focus on bonding with their child [6].

## Conclusions

There is still little knowledge about cancer during pregnancy, even less so with regards to the psychological impact of the diagnosis on the mother’s functioning and of the long-lasting effects that this stressful life event might have on the development of the attachment system.

It seems imperative to conduct an in-depth analysis of the psychological processes and the development of the mother-child relationship in cases of cancer during pregnancy, in order to develop an integrative model of care which aims for the best possible medical and psychological outcomes.
